# Endocannabinoid System Components as Potential Biomarkers in Psychiatry

**DOI:** 10.3389/fpsyt.2020.00315

**Published:** 2020-04-27

**Authors:** Francisco Navarrete, María Salud García-Gutiérrez, Rosa Jurado-Barba, Gabriel Rubio, Ani Gasparyan, Amaya Austrich-Olivares, Jorge Manzanares

**Affiliations:** ^1^Instituto de Neurociencias, Universidad Miguel Hernández-CSIC, Alicante, Spain; ^2^Red Temática de Investigación Cooperativa en Salud (RETICS), Red de Trastornos Adictivos, Instituto de Salud Carlos III, MICINN and FEDER, Madrid, Spain; ^3^Instituto de Investigación i+12, Hospital Universitario 12 de Octubre, Madrid, Spain; ^4^Servicio de Psiquiatría, Hospital Universitario 12 de Octubre, Madrid, Spain; ^5^Departamento de Psicología, Facultad de Educación y Salud, Universidad Camilo José Cela, Madrid, Spain; ^6^Department of Psychiatry, Complutense University of Madrid, Madrid, Spain

**Keywords:** endocannabinoid system, cannabinoid receptor (CB1r, CB2r), endocannabinoid, biomarker, sychiatry, diagnosis, treatment

## Abstract

The high heterogeneity of psychiatric disorders leads to a lack of diagnostic precision. Therefore, the search of biomarkers is a fundamental aspect in psychiatry to reach a more personalized medicine. The endocannabinoid system (ECS) has gained increasing interest due to its involvement in many different functional processes in the brain, including the regulation of emotions, motivation, and cognition. This article reviews the role of the main components of the ECS as biomarkers in certain psychiatric disorders. Studies carried out in rodents evaluating the effects of pharmacological and genetic manipulation of cannabinoid receptors or endocannabinoids (eCBs) degrading enzymes were included. Likewise, the ECS-related alterations occurring at the molecular level in animal models reproducing some behavioral and/or neuropathological aspects of psychiatric disorders were reviewed. Furthermore, clinical studies evaluating gene or protein alterations in *post-mortem* brain tissue or *in vivo* blood, plasma, and cerebrospinal fluid (CSF) samples were analyzed. Also, the results from neuroimaging studies using positron emission tomography (PET) or functional magnetic resonance (fMRI) were included. This review shows the close involvement of cannabinoid receptor 1 (CB1r) in stress regulation and the development of mood disorders [anxiety, depression, bipolar disorder (BD)], in post-traumatic stress disorder (PTSD), as well as in the etiopathogenesis of schizophrenia, attention deficit hyperactivity disorder (ADHD), or eating disorders (*i.e.* anorexia and bulimia nervosa). On the other hand, recent results reveal the potential therapeutic action of the endocannabinoid tone manipulation by inhibition of eCBs degrading enzymes, as well as by the modulation of cannabinoid receptor 2 (CB2r) activity on anxiolytic, antidepressive, or antipsychotic associated effects. Further clinical research studies are needed; however, current evidence suggests that the components of the ECS may become promising biomarkers in psychiatry to improve, at least in part, the diagnosis and pharmacological treatment of psychiatric disorders.

## Introduction

Psychiatric disorders are one of the main causes of disability in the general population (1). According to a recent estimation, psychiatric disorders account for 32.4% of years lived with disability (YLDs) and 13% of disability adjusted life-years (DALYs), leading the global burden of disease (2). Despite this, we still have a great lack of knowledge about its neurobiological basis, and clinically applicable biomarkers have been elusive. During the last decades, an increasing effort has been made in the search of biomarkers in psychiatry to help in the diagnosis and prediction of disease progression or treatment response. However, a clinical biomarker should be validated, sensitive, specific, feasible, and easily reproducible, characteristics that make difficult the implementation in this field (3–5).

The endocannabinoid system (ECS) components (receptors, ligands, synthesizing and degrading enzymes) have gained a special interest because of their critical neuromodulatory involvement in a plethora of functional mechanisms in the central nervous system (CNS), including emotional regulation, motivational behavior, and cognitive function (6, 7). The wide distribution of ECS in the brain, together with the effects derived from its pharmacological modulation on mood or cognition with exogenous cannabinoid compounds, mainly those contained or derived from the *Cannabis sativa* plant, suggests that the identification of the functional role of ECS elements in certain psychiatric disorders could be a breakthrough to improve diagnosis and treatment (8–11).

Therefore, this review summarizes the findings regarding the potential involvement of ECS components as biomarkers, mainly in terms of the discovery of new therapeutic approaches, but also from the point of view of its diagnostic, prognostic and predictive application. For that purpose, studies on animal models and patients have been collected focusing on the most prevalent psychiatric conditions, including anxiety disorders (3.8%) (12), depressive disorders (3.4%) (12), schizophrenia (0.3%) (12), bipolar disorder (0.6%) (12), post-traumatic stress disorder (7.8%) (13), attention-deficit hyperactivity disorder (2.2%) (14), and eating disorders (0.2%) (12).

## A Brief Overview of the Endocannabinoid System Components

**Figure 1:**
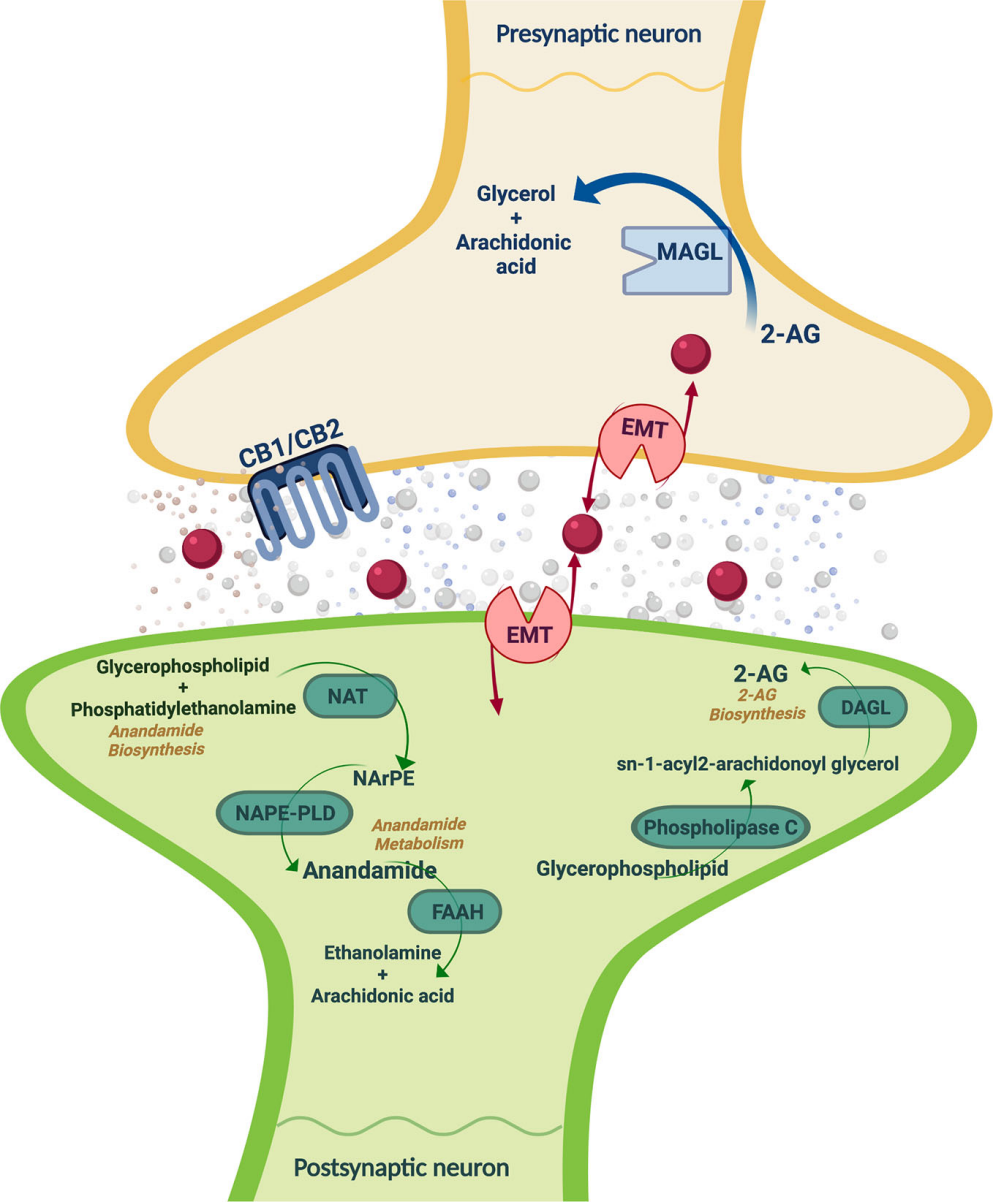
Schematic representation of the main ECS components, including the metabolizing routes of the eCBs. CB1/CB2, cannabinoid receptors 1 and 2; 2-AG, 2-arachidonoylglycerol; FAAH, fatty acid amide hydrolase; MAGL, monoacylglycerol lipase; DAGL, EMT: endocannabinoid membrane transporter; NAT, N-acyl transferase; NArPE, N-arachidonoyl phosphatidylethanolamine; NAPE-PLD, N-acylphosphatidylethanolamine specific phospholipase D; DAGL, diacylglycerol lipase. Image created with BioRender.

ECS regulates a number of physiological functions and mediates the crosstalk between different neurotransmitter systems, therefore representing a key player in the control of behavioral responses (15, 16). ECS is a ubiquitous lipid signaling system distributed throughout the organism that participates in multiple intracellular signaling pathways (17, 18). Cannabinoid receptors, endogenous ligands or endocannabinoids (eCBs), and their synthesizing and degrading enzymes are the main components of the ECS (**Figure 1**) present in the central and peripheral nervous system (15, 19) and in many other peripheral tissues regulating distinct functions (20).

The CB1 receptor (CB1r) is the most abundant G protein-coupled receptor in the brain (21). Physiological actions of endocannabinoids in the CNS are mediated by the activation of CB1r (22). Their expression in the CNS is widespread and heterogeneous and has crucial roles regulating brain function and disease processes (23–25). CB1r is abundant in the basal ganglia, cerebellum, in corticolimbic regions including the prefrontal cortex (PFC), nucleus accumbens (Nac), and hippocampus (Hipp), and in brain areas related to stress responses, such as the central amygdala (Amy) and the paraventricular nucleus (PVN) of the hypothalamus (Hyp) (21, 26, 27). Furthermore, CB1r is also located in terminals of peripheral neurons and glial cells, as well as in the reproductive system (*i.e.* uterus, ovary, testis, prostate), some glandular systems (adrenal gland), adipose tissue, heart, liver, lung, bone marrow, thymus, and the microcirculation (20, 26, 28–33).

CB2 cannabinoid receptor (CB2r) was initially considered as a peripheral cannabinoid receptor due to its high expression in the rat spleen (34) and leukocyte subpopulation in humans (32), participating in the regulation of the immune system (35). The first findings identified the presence of CB2r in the CNS only under pathological conditions such as in senile plaques in Alzheimer's disease (36), activated microglial cells/macrophages in multiple sclerosis, spinal cord in amyotrophic lateral sclerosis (37) and in the vicinity of tumors (38). However, Van Sickle and colleagues revealed that CB2r is expressed in neurons of the brainstem of mice, rats, and ferrets under normal conditions (39). This finding was key to increase the interest of CB2r in the regulation of brain function. Different studies identified CB2r in several brain regions including the frontal cortex, striatum, basal ganglia, Amy, Hipp, and the ventral tegmental area (VTA) (40–44). Interestingly, in some of these brain regions, CB2r was detected not only in the microglia (45) but also in the neurons (44, 46, 47).

The eCBs are lipid messengers acting as paracrine, autocrine, and probably endocrine mode, because their lipid nature allows them to diffuse and cross membranes (15, 17, 18, 48, 49). eCBs are agonists of CB1r and CB2r that are not accumulated in secretory vesicles but rather synthesized under tonic or phasic (on demand) modes, and released to the extracellular space following physiological and pathological stimuli (50). The two main eCBs are derivatives of polyunsatured fatty acids, N-arachidonoylethanolamine (anandamide, AEA) (51), and 2-arachidonoylglycerol (2-AG), being the most abundant eCBs in the brain (52). Firstly, AEA synthesis is produced by the N-acylphosphatidylethanolamine specific phospholipase D (NAPE-PLD) that hydrolyzes N-arachidonoyl phosphatidylethanolamine localized in cell membranes (49, 53). The AEA half-life is very short because of its quick uptake by a high affinity AEA membrane transporter distributed in the neurons and glia (54). AEA is inactivated by fatty acid amide hydrolase (FAAH) present in many organs and in the brain at postsynaptic location (55, 56). FAAH is a serine-hydrolase enzyme bound to intracellular membranes that metabolizes AEA into arachidonic acid and ethanolamine (57). Secondly, 2-AG participates in the CB1r-dependent retrograde signaling and is an intermediate metabolite for lipid synthesis providing arachidonic acid for prostaglandin synthesis (57). Neuronal membrane depolarization or the activation of Gq protein-coupled receptors (GPCRs) triggers the synthesis of 2-AG (49). The diacylglycerol precursors come from the hydrolysis of membrane phosphatidylinositol by phospholipase C, *β* or *δ*. The degradation of these precursors by diacylglycerol lipases (DAGL-*α* and DAGL-*β*) drives 2-AG synthesis (58, 59). The DAGL*α* isoform synthesizes the greatest amount of 2-AG, whereas DAGL*β* synthesizes 2-AG under certain circumstances (54). Monoacylglycerol lipase (MAGL) is a serine-hydrolase enzyme mainly found in presynaptic terminals that catalyzes 2-AG into arachidonic acid and glycerol (55, 60). Also, the *α*/*β*-hydrolase domain 6 (ABHD6) and domain 12 (ABHD12) degrade 2-AG (49, 57).

## The Endocannabinoid System in Psychiatry: Searching for Potential Biomarkers

The ECS is one of the most widely distributed neurotransmitter systems in the human brain, with a critical neuromodulatory role that motivates the interaction with other neurotransmitter and neurohormonal systems (61). Accumulating evidence points out the pivotal role of the ECS in the regulation of cognitive and behavioral functioning, suggesting its therapeutic potential in psychiatry (9, 11, 62). Furthermore, it is worth to mention that psychiatric disorders are accompanied by disturbances in the ECS components, as detailed below. Taken together, these facts suggest the potential usefulness of cannabinoid receptors, endocannabinoid ligands and degrading or synthesizing enzymes as biomarkers to move towards improved diagnostic criteria and therapeutic approaches in psychiatry.

The literature review consisted of an exhaustive search for scientific information in the Medline database (PubMed), which was always focused on the following ECS components as potential biomarkers in psychiatry: CB1r, CB2r, AEA, 2-AG, FAAH and MAGL. A total of seven search boxes were employed according to the total of psychiatric conditions included in the review: anxiety, depression, schizophrenia, bipolar disorder, post-traumatic stress disorder, attention-deficit and hyperactivity disorder, and eating disorders. These terms were combined with the term ‘cannabinoid’ by the Boolean operator ‘AND’. All the results for each search were critically analyzed by the authors to decide the inclusion or exclusion of each reference according to the adequacy of its content with the subject matter of the study. Finally, no PubMed filters were applied to maximize the selection of all the available and appropriate information.

### Anxiety Disorders

According to the Diagnostic and Statistical Manual of Mental Disorders (DSM-5), anxiety disorders share features of excessive fear and anxiety and related behavioral disturbances. Fear is the emotional response to real or perceived imminent threat, whereas anxiety is an emotional anticipatory response to future potential threatening or stressful situations, triggering symptoms of negative affective, somatic, behavioral and cognitive components (63). The ECS plays a prominent role in the stress response and anxiety, as it is widely documented mainly by animal studies (64–67). However, our knowledge on the precise molecular mechanisms of the ECS signaling in humans is insufficient (68, 69). In the last years, compelling evidence for the involvement of ECS in anxiety has been accumulated that suggests new therapeutic leads through the discovery of potential biomarkers.

#### Animal Studies

A large body of literature supports the involvement of CB1r as a potential biomarker in anxiety disorders (70–72). CB1r is widely distributed in brain areas associated with emotional regulation and stress responsiveness such as PFC, Hipp, Amy, and Hyp (19). Previous pharmacological studies evaluated the effects of different cannabinoid compounds after either systemic or intracerebral administration in rodents exposed to several animal models of anxiety (73, 74). In addition, it is important to highlight the pivotal role of CB1r in the effects of anxiolytic drugs such as benzodiazepines. Indeed, our group demonstrated that the CB1r antagonist, AM251, completely abolished the anxiolytic effects and significantly reduced the amnesic and the sedative actions induced by alprazolam (75). A very similar result was recently obtained regarding the AM251-induced blockade of the anxiolytic effects of alprazolam (76). On the other hand, the enhancement of CB1r-mediated endocannabinoid function increases the anxiolytic action of diazepam (77).

Accumulated evidence points out that CB1r manipulation produces a bidirectional effect on anxiety-related behavior (78, 79). CB1r activation decreases anxiety at lower doses (80), whereas anxiogenic effects occur at higher doses or after CB1r blockade (70, 81–87). However, several factors could modify this general assumption such as regional endogenous tone, age, sex, species differences, type of test, previous exposure to stressful situations, or dosage of cannabinoid receptor agonists or antagonists. In addition, the underlying mechanisms involved in the bidirectional effects of CB1r pharmacological modulation remain poorly understood. Among the available evidences addressing this aspect, one study revealed that CB1r in the cortical glutamatergic neurons mediates the anxiolytic effect of CP-55,940 cannabinoid agonist at low doses, whereas anxiogenic actions of higher doses are related with CB1r and GABA_B_ receptors in GABAergic terminals (88). A growing body of evidence also suggests that the anxiogenic effects of moderate to high cannabinoid doses appear to be mediated by the interaction between endocannabinoid and endovanilloid systems, specifically through the activation of transient receptor potential cation channel subfamily V member 1 (TRPV1) vanilloid receptors (89). In this regard, the combination of high WIN-55,212 doses in the dorsolateral periaqueductal gray matter (dlPAG) with the TRPV1 antagonist capsazepine abolished the anxiogenic effect (90). Furthermore, the anxiolytic effects of high doses of the cannabinoid agonist ACEA combined with an antagonist of TRPV1 in the rat prelimbic medial prefrontal cortex (PL) suggested the critical interaction between both systems (91). Moreover, the co-administration of intra-dlPAG AEA at higher doses with a nitric oxide (NO) scavenger (carboxy-PTIO) restored the anxiolytic profile, leading to the hypothesis that the increase in anxiety-like behavior mediated by TRPV1 receptors is due to subsequent NO formation (92).

Deletion of *CNR1* gene in mice (CB1^−/−^ mice) has been another important tool to elucidate the role of this cannabinoid receptor in anxiety. Many studies have demonstrated the clear anxiety-like behavior of male CB1^−/−^ mice (70–72, 83, 93, 94), although there are some negative results (95). Among the multiple mechanisms involved in the anxious phenotype shown by CB1^−/−^ mice, significant age-dependent alterations in the metabolism of endocannabinoids could be pointed out (96). Interestingly, CB1^−/−^ female mice do not have an anxious phenotype in comparison with female wild-type (WT) subjects. This finding supports an interaction between sex and the ECS at early stages of development that is critical for establishing adult anxiety-like behavior (97). Indeed, these sex-specific effects were also described under pharmacological blockade of CB1r (98). Furthermore, our group described that the effects of the anxiolytic drugs bromazepam and buspirone were missing in CB1^−/−^ mice (99), suggesting a critical role of CB1r that was related with the control of GABAergic responses mediated by GABA_A_ and GABA_B_ receptors (100).

Recent studies provide relevant information regarding the specific brain regional involvement of CB1r-mediated anxiolytic actions. In this sense, the intra-dlPAG administration of AEA, ACEA (selective CB1r agonist) or AM404 (AEA reuptake inhibitor) induced anxiety-like responses that were blocked by AM251 (CB1r antagonist) (101). Similarly, AEA-mediated CB1r activation produces anxiolytic-like actions in the dlPAG employing a panic-like animal model (102, 103) or the Vogel conflict test (104). In addition, facilitation of 2-AG-mediated signaling in the dorsomedial hypothalamus (DMH) significantly reduced panic-like responses in Wistar rats, an effect that was reversed by the CB1r antagonist AM251 (105). Furthermore, activation of CB1r by 2-AG in the basolateral amygdala (BLA) has a critical role in the effects of stress-induced glucocorticoid release on suppression of synaptic GABAergic inhibition (106). Interestingly, pharmacologically-induced elevations of AEA or 2-AG in the BLA decrease anxiety in the elevated plus maze (EPM) test under conditions of low emotional arousal while are ineffective when the level of emotional arousal increased (107). Moreover, electron microscopy revealed CB1r expression in the rat lateral habenula (LHb), mediating the actions of increased 2-AG levels after acute stress exposure, while its blockade by SR141716 (rimonabant) significantly reduced anxiety-like behavior (108). In another study, WIN-55,212 was locally administered in the lateral septum (LS) of male Wistar rats, producing a CB1r-mediated anxiogenic response in the EPM paradigm since AM251 blocked this effect (109). Also, the role of CB1r functional manipulation in anxiety behavior regulation and the effects on subsequent signaling pathways in relevant corticolimbic areas such as PFC, AMY, NAc, and Hipp (110–116) have been evaluated.

A better understanding of the functional connections of the ECS with other neurotransmitter or neurohormonal systems is relevant to understand the role of ECS components as potential biomarkers in psychiatry. According to previous studies, CB1r is located in the locus coeruleus (LC) and in the dorsal raphe nucleus (DRN), and it regulates noradrenaline (NA) and serotonin (5HT) release, respectively, by the modulation of GABAergic and glutamatergic terminals (117, 118). In addition, the dopaminergic and opiodergic systems of the Amy may also be involved in the anxiolytic-like effects induced by the activation of CB1r (119, 120). Furthermore, the involvement of the ECS in the regulation of the hypothalamus–pituitary–adrenal (HPA) axis after stress exposure attracted special attention in the last years (121). Gray and cols. recently found that the stress-related neuropeptide corticotropin-releasing hormone (CRH), acting through the CRH type 1 receptor (CRHR1), reduces AEA levels in the PFC and the Amy by increasing the hydrolysis of FAAH and by increasing 2-AG levels. These data suggest that stress-related elevations in CRH signaling induce persistent changes in eCB function, impairing its tonic regulation on stress and enhancing anxiety responses (122, 123).

Genetic studies pointed out interesting results regarding the involvement of polymorphisms or epigenetic modifications of CNR1 as susceptibility/risk biomarkers to develop anxiety disorders. Lazary and cols. analyzed the interaction of the promoter regions of the serotonin transporter (5HTT; SLC6A4) and CNR1 genes on anxiety. Specific constellations of CB1r and 5HTT promoters were closely associated with high or low synaptic 5HT concentrations, which could result critically in the vulnerability to experience an anxiety disorder (124). Hay and cols. employed CRISPR/CAS9 technology to disrupt a highly conserved regulatory sequence (ECR1) of the gene encoding CB1r (CNR1). This manipulation significantly reduced CNR1 expression in the Hipp, but not in the Hyp, and induced a sex-dependent anxiogenic effect (125). In addition, a connection between ECS and epigenetic mechanisms was proposed. The exposure to immobilization stress increases anxiety-like behavior, an effect blocked by histone deacetylase (HDAC) inhibitors. Interestingly, the CB1r antagonist rimonabant attenuated the anxiolytic-like effects of the HDAC inhibitors, suggesting an association between epigenetic mechanisms and ECS signaling (126). Furthermore, in mice exposed to a chronic unpredictable stress (CUS) there were reduced levels of histone H3K9 acetylation (H3K9ac) associated with CB1r encoding gene (127).

Since the direct pharmacological modulation of CB1r has provided some disappointing results, in recent years much attention has been paid to the therapeutic role of functional manipulation of the endogenous cannabinoid ligands AEA and 2-AG by inhibiting enzymatic degradation (FAAH and MAGL, respectively) or blocking reuptake (128–132). AEA plays a crucial role in emotional control (133). Inhibition of its degradation by FAAH or its reuptake induces a robust anxiolytic effect (134–142). Indeed, stress exposure induces anxiety-like behavior and reduces AEA brain levels (143) by increasing FAAH activity in the Amy (144), a brain region closely involved in AEA-mediated emotional regulation (145). According to the effects observed in FAAH *knockout* mice (FAAH^−/−^ mice), as well as with the administration of URB597 (FAAH inhibitor), preservation of CB1r function regulating GABA transmission in the striatum may be one the mechanisms involved in the anxiolytic actions of FAAH inhibition (146, 147). Environmental experimental conditions are critical to observe the anxiolytic effect of FAAH inhibition, only present under high stressful or aversive stimuli (148). Interestingly, the dual blockade of FAAH and TRPV1 represents another therapeutic approach to reduce anxiogenic behavior (149). In addition, co-administration of an ineffective dose of URB597 with an ineffective dose of diazepam led to a synergistic anxiolytic action (77).

The endocannabinoid 2-AG also presents a close involvement in emotional regulation linked with signaling in hippocampal glutamatergic neurons (150). In the last years, several evidences support the anxiolytic actions associated with the inhibition of 2-AG enzymatic degradation by means of MAGL (151–153). A link with the HPA axis has been proposed, since the elevation of 2-AG levels was accompanied by a dramatic increase in plasma corticosterone, effect that is probably mediating its anxiolytic actions (154). In addition, increased 2-AG levels in the NAc of mice previously exposed to chronic social defeat stress are associated with an anxiolytic effect and the enhancement of synaptic plasticity (155). Furthermore, the enhancement of 2-AG levels in the dlPAG by the local injection of 2-AG or the hydrolysis inhibitor, URB602, prevented NMDA-induced panic-like response in Wistar rats (156). Interestingly, genetic deletion of MAGL in mice induced an anxiety-like phenotype (157), whereas mice lacking DAGL*α* showed a high anxiety-like phenotype, strengthening the critical involvement of 2-AG in emotional regulation (158, 159).

Another relevant endocannabinoid biomarker is the CB2r. The first studies demonstrating the role of this receptor in the regulation of anxiety-like behavior were performed in our laboratory employing transgenic animals overexpressing CB2r in the brain (CB2xP mice). Increased expression of CB2r was significantly correlated with reduced anxiogenic-related behaviors. Interestingly, CB2xP mice presented an impaired HPA-axis response to restraint stress, as well as increased GABA_A_*_α_*_2_ and GABA_A_*_γ_*_2_ gene expression probably accounting for the lack of anxiolytic action of alprazolam in these animals (160). Furthermore, a pharmacological approach to evaluate acute and chronic effects of the activation (JWH133, CB2r selective agonist) or blockade (AM630, CB2r selective antagonist) of CB2r revealed opposite effects. Importantly, chronic CB2r blockade induced a significant anxiolytic effect that was associated with an upregulation of CB2r, GABA_A_*_α_*_2_ and GABA_A_*_γ_*_2_ in the cortex and the amygdala (160). In line with these results, the acute activation of CB2r by the administration of *β*-caryophyllene (BCP) induced an anxiolytic effect that was completely abolished by AM630-mediated CB2r blockade (161). Recently, Robertson and cols. described that CB2r gene expression is rapidly increased in the Hipp after social stress exposure (social defeat) (162). Genetic manipulation experiments allowed the deepening in the cell-specific functional involvement of CB2r in the Hipp, dissecting the effects of CB2r gene expression disruption in hippocampal neurons or microglia on the regulation of anxiety behavior (163). Moreover, the functional role of CB2r in VTA dopaminergic neurons was also explored. Surprisingly, deletion of CNR2 in VTA dopaminergic neurons induced a very significant anxiolytic effect (47).

#### Human Studies

In 1981, Fabre and McLendon published the first evidences regarding the anxiolytic properties of cannabinoid compounds. In this study, the synthetic cannabinoid nabilone was administered to 25 patients, producing a significant improvement in anxiety (164). Nowadays, there is a large body of evidence regarding cannabis consumption and regulation of anxiety behavior (165), although the underlying mechanisms are poorly understood. A recent study addressed this issue by combining fMRI and positron emission tomography (PET) in 14 patients following an oral dose of delta-9-tetrahydrocannabinol (THC) while they were performing a fear-processing task. The results suggested that the acute effects of cannabis on anxiety in males are mediated by the modulation of amygdalar function by THC and the extent of these effects are related to local availability of CB1r (166). On the other hand, several clinical trials using rimonabant to treat obesity showed psychiatric side effects such as increased anxiety behavior, depression or even suicidality (167). In spite of the presence of important confounding factors that probably were not appropriately taken into consideration (*e.g.* psychiatric comorbidity in obese patients), rimonabant was withdrawn from the market, and the enthusiasm in its therapeutic usefulness significantly decreased. Interestingly, a recent report suggested that rimonabant increases anxiety only under an aversive/anxiogenic situation (public speaking), without modifying baseline anxiety behavior (168). Alternative pharmacological approaches to modulate CB1r are now under investigation. Neutral antagonists, peripherally restricted ligands, and allosteric modulators may provide promising results [for a recent review (169)].

The elucidation of genetic variations of different endocannabinoid components involved in the vulnerability to develop anxiety-related disorders has recently gained great interest. In this regard, Gonda and cols. evaluated the interaction between four categories of stressful life events and specific genetic variations in the CNR1 rs7766029 polymorphism, for the development of depression and anxiety. The results suggested that CNR1 rs7766029 interacted significantly with financial but not with other types of life events to increase the vulnerability to develop depression and anxiety (170). In addition, allelic variants of the gene encoding FAAH have been involved in the regulation of anxiety-related behaviors. First, the disturbances of FAAH genetic variation in AEA hydrolysis appear related with alterations in frontolimbic circuits (171), with an age-dependent effect accounting for differences between the adolescence and childhood life stages (172). Second, an interaction between genetic variations of FAAH and corticotropin-releasing hormone receptor type 1 (CRHR1) has been described in relation with the risk to develop anxiety disorders (173, 174). Third, reduced FAAH activity in patients carrying the A allele of the FAAH rs324420 (C385A) polymorphism significantly increases the vulnerability to develop anxiety and depression when exposed to repetitive childhood trauma (175). Moreover, a functional variant of gene encoding CB2r (Cnr2) appears to interact with FAAH gene, increasing the sensitivity for childhood trauma when both are dysfunctional (176).

### Depressive Disorders

Major depressive disorder (MDD) has been one of the leading causes of years lived with disability (YLD) during the last three decades (1). According to the World Health Organization estimation for 2015, the number of people living with depression in the world is 322 million, and it is a major contributor to suicide deaths (177). DSM-5 states that the common feature of depressive disorders is the presence of sad, empty, or irritable mood, accompanied by somatic and cognitive changes that significantly affect the individual's capacity to function. MDD is being characterized by distinct changes in affect, cognition, and neurovegetative functions with episodes lasting for at least 2 weeks. Additionally, five or more symptoms have to be present during the same episode, with at least one of the symptoms being either depressed mood or anhedonia (63). Nowadays, pharmacological treatment of MDD entails relevant limitations such as delayed onset of antidepressive actions and appearance of important side effects. The limited success of drug discovery in the context of depression is ultimately linked to an inadequate understanding of the underlying biology of this disorder. In this sense, there is evidence to suggest that the ECS is impaired in MDD providing a unique opportunity to identify potential diagnostic and therapeutic biomarkers.

#### Animal Studies

Martin and cols. employed the CB1^−/−^ mice and exposed them to the CUS procedure. Their findings showed that CB1^−/−^ were more vulnerable to CUS-induced depressive-like responses and presented an increase susceptibility to develop anhedonia (94). Some years later, it was shown that the increased despair behavior in CB1^−/−^ mice was critically associated with down-regulated brain-derived neurotrophic factor (BDNF) levels in the Hipp. Also, local administration of BDNF in the Hipp of these animals reversed the depressive-like phenotype (178). A complete genetic screening by mRNA microarray hybridization revealed a differential gene expression pattern related to the high depressive-like behavior of CB1^−/−^ mice at basal conditions (179). According to the results derived from the studies employing CB1^−/−^ mice, it was proposed that CB1^−/−^ mice could represent a validated and appropriated model to evaluate depressive-like disorders (180).

In the tail suspension test (TST) and forced swimming test (FST), acute AM251 injection induced an antidepressant effect, decreasing the immobility time in both behavioral paradigms (181). Similar results were obtained by both the acute and chronic administration of rimonabant in Wistar rats and BALB/c mice employing the FST and the chronic mild stress (CMS) paradigms, respectively (182). However, other results reveal that the activation of CB1r mediates antidepressant effects (183–187), and even that chronic rimonabant administration produces a depressogenic effect (188). Interestingly, McLaughlin and cols. showed that CB1r located in the dentate gyrus of the Hipp was responsible for the antidepressant effects of the CB1r agonist HU-210 (189). In addition, a very recent study elegantly discovered a circuit-specific CB1r-mediated modulation of glutamatergic transmission that shapes the information flow from BLA to the NAc (190). In this study, the authors consider if the reduction of CB1r in the NAc may be used as a biomarker for MDD diagnosis and point out that this aspect needs to be further determined by evaluation of CB1r levels in the NAc of MDD patients (190).

The evaluation of ECS components disturbances in animal models of depressive disorders provided relevant information. Hill and cols. showed that male Long–Evans rats exposed to the CUS presented increased CB1r binding site density in the PFC while decreased in the Hipp, Hyp and Nac, and lower levels of AEA were found in all these brain regions (191). Furthermore, sex-dependent effects of CUS were analyzed in Sprague-Dawley rats, obtaining lower and higher CB1r protein expression in males and females, respectively, whereas increased FAAH levels were present in both sexes (192). In addition, further studies employing the CUS procedure specifically focused on CB1r-mediated signaling, revealing significant loss of function disturbances in the NAc (193) and in the LHb (194). Moreover, apart from stress-related animal models, Flinders Sensitive Line (FSL) or Wistar Kyoto (WKY) rats are well-known genetic rat models of depression that were recently used to exhaustively analyze disturbances in different components of the ECS in specific brain regions and plasma (195, 196).

Enhancement of endocannabinoid signaling has been postulated as a new promising pharmacological strategy in the treatment of stress-related disorders (*e.g.* anxiety or depression) (197). Accordingly, a significant reduction in depressive-like behavior was found after the administration of the FAAH inhibitors URB597 (196, 198, 199) or PF3845 (200). In addition, the inhibition of MAGL by the administration of JZL194 also yielded similar antidepressant effects in the CUS animal model of depression. Interestingly, JZL194-mediated effects may be related with an enhancement of adult neurogenesis and long-term synaptic plasticity in the dentate gyrus of the Hipp, probably activating mTOR signaling pathway (201). Furthermore, a recent study evaluated the effects of JZL195, a dual inhibitor of FAAH and MAGL, in WKY rats. JZL195 elevated the endocannabinoids and BDNF levels in the ventral striatum and reduced the depressive-like phenotype in female WKY rats (202).

In spite of the limited available results, CB2r is also critically involved in emotional regulation (203). Probably, the first evidence suggesting the role of CB2r in depression was a significant reduction of these receptors in the striatum, midbrain, and Hipp in an animal model of depression (204). Afterwards, a study showed the antidepressant effects of the CB2r-seletive agonist GW405833 in rats (205). Interestingly, our group further evaluated CB2r involvement in depressive-like behavior regulation using genetic and pharmacological approaches. Mice overexpressing CB2r (CB2xP) presented decreased depressive-like behaviors under basal conditions or after the exposure to a CUS procedure. In addition, the chronic administration of AM630 blocked the CUS-induced depressogenic effect in stressed mice, effect associated with an upregulation of CB2r and BDNF in the Hipp (43). Recently, similar results were obtained by CB2r functional activation through the administration of the CB2r agonists JWH133 (206) and *β*-caryophyllene (207). Furthermore, the specific deletion of CB2r in midbrain DA neurons in DAT-Cnr2 conditional knockout (cKO) mice significantly increased depressive-like behavior (47).

Crosstalk of ECS with other neurotransmitter or neurohormonal systems plays a pivotal role in the effects produced by antidepressant drugs. In this regard, interactions with the serotonergic system represent a critical point due to the widely recognized clinical therapeutic usefulness of antidepressants targeting serotonin (*e.g.* serotonin selective reuptake inhibitors, SSRIs). SSRIs fluoxetine and escitalopram modify the concentrations of different ECS components under basal conditions (208–210) or in an animal model of depression (211). Furthermore, low doses of WIN-55,212 produced antidepressant-like actions that appeared to be mediated by 5HT (212), and CB1^−/−^ mice have decreased levels of 5HT transporter (5HTT) (213). Moreover, co-administration of a subeffective dose of fluoxetine potentiated the effect of subeffective doses of AEA, AM404 or URB597 (214). In addition, ECS also interacts with other systems involved in emotional and stress regulation such as the HPA axis (215), glutamatergic (216), opioidergic (217), and cholinergic (218) systems.

On the other hand, it is relevant to highlight that nonpharmacological approaches such as repeated transcranial magnetic stimulation (rTMS) improve depressive-like behavior, at least in part, by modulating the ECS. Recent studies performed in rodents exposed to CUS and subsequently treated with rTMS revealed that: 1) rTMS increases BDNF production and hippocampal cell proliferation to protect against CUS-induced changes through its effect on CB1r (219); 2) rTMS antidepressive effects are at least partly mediated by increasing hippocampal 2-AG and CB1 receptor expression levels (220); and 3) high-frequency rTMS induces its antidepressant effect by upregulating DAGL*α* and CB1r (221). In addition, electroconvulsive therapy (ECT) significantly reduced AEA content and FAAH activity in the PFC of Sprague-Dawley rats, as well as decreased and enhanced binding site density of the CB1r in the PFC and Amy, respectively (222).

#### Human Studies

Besides the preclinical clues supporting the critical role of ECS in depression, currently there is a broad body of evidence available from clinical studies. Among them, those evaluating alterations in different ECS components in *post-mortem* brain tissue or plasma samples have provided compelling results. The first evidence revealed that CB1r protein expression was decreased in the anterior cingulate cortex (ACC) of patients with major depression (223). Furthermore, Choi and cols. showed that CB1r mRNA levels were higher in the PFC of major depression patients (224). However, in a recent study a lack of CB1r protein expression differences was found between depressive subjects and paired control patients (225).

In the last years, an increasing effort has been made to elucidate the alterations of ECS components (mainly the endocannabinoids AEA and 2-AG) in blood samples of patients with depression, to identify possible trait, prognosis or monitoring biomarkers that could improve the therapeutic approach. In a cohort of 28 women with diagnostic criteria for clinical depression and without medication, serum 2-AG content was significantly decreased, and this decrease was negatively correlated with duration of the depressive episode (226). Similarly, basal serum concentrations of AEA and 2-AG were significantly lower in women with nontreated major depression, and the exposure to a stressful situation significantly increased 2-AG concentrations without modifying AEA (227). However, another study described increased plasma concentrations of both AEA and 2-AG in depressed patients, and the elevation of 2-AG was significantly associated with SSRI antidepressant therapy (228). Interestingly, the antidepressant-related effects or physical exercise on eCBs levels were also analyzed. Intense exercise in control healthy patients induced a significant increase in AEA serum levels that was correlated with higher BDNF levels, whereas 2-AG concentrations remained stable (229). On the contrary, moderate exercise in women with MDD produced significant elevations in AEA but not in 2-AG, although both eCBs presented significant moderate negative associations between serum changes and mood states (230). Finally, ECT significantly elevated AEA and 2-AG levels in the cerebrospinal fluid (CSF) of patients with major depression (231).

The ECS-related polymorphic gene variant study results are relevant because of the potential diagnostic and therapeutic implications. Regarding the CNR1 and the single nucleotide polymorphism (SNP) rs1049353 (G1359A) that may contribute to the susceptibility to mood disorders (232), G-allele has been associated with higher depressive-related symptomatology (233) and increased risk of antidepressant treatment resistance in women with comorbid anxiety disorder (234). However, it provides a better response to citalopram in male depressive patients (235), whereas A-allele decreased risk to develop depression because of childhood physical abuse (236). Furthermore, several CNR1 polymorphisms appeared to be related with high neuroticism and low agreeableness personality traits, increasing the risk to develop depression (237). On the other hand, the presence of 2 long alleles of the polymorphic triplet (AAT)n of CNR1 gene was associated with reduced prevalence of depression in Parkinson's disease patients (238). In addition, the minor C allele of the CNR1 rs2023239 polymorphism may confer a protective effect against lifetime development of MDD in methadone-maintained patients (239). Despite the previous findings, a recent meta-analysis points out that CNR1 rs1049353 or AAT triplet repeat polymorphism had no association with susceptibility to depression (240).

Other relevant gene polymorphisms of the ECS are those related with FAAH and CB2r. First, variants of the FAAH gene may be related with susceptibility to mood disorders such as major depression (232). In fact, genetically reduced FAAH activity in A allele carriers of FAAH rs324420 (C385A) polymorphism constitutes a risk factor to develop anxiety and depression in patients exposed to repetitive childhood trauma. Interestingly, the authors noted that this genotype could entail pharmacogenomic consequences, namely ineffectiveness or adverse effects of FAAH inhibitors in this subpopulation (175). Second, polymorphisms of CNR2 were first studied by Onaivi and cols. in Japanese depressed patients, revealing a high incidence of Q63R but not H316Y polymorphism (204, 241). Recently, the R allele of Q63R CNR2 polymorphism, together with the A allele of FAAH C385A polymorphism were associated with increased sensitivity for childhood trauma and subsequent expression of anxious and depressive phenotypes (176). Finally, according to the previously mentioned recent meta-analysis performed by Kong and cols., CNR2 rs2501432 polymorphism might be closely associated with depression (240).

### Schizophrenia

According to the DMS-5 schizophrenia is a psychotic disorder associated with a myriad of signs including positive symptoms (delusion, hallucinations, disorganized speech or grossly disorganized or catatonic behavior), negative symptoms (lack of motivation and social withdrawal), and cognitive symptoms (reduced attention and altered speech) (63, 242, 243). An extensive body of literature supports the role of ECS in schizophrenia neuropathology, a fact that is mainly sustained by the psychotic effects derived from cannabis consumption and attributed to the exogenous cannabinoid THC (244). Therefore, a great interest has been posed in the identification of specific biomarkers related with ECS functioning for preventive, diagnostic, or therapeutic purposes.

#### Animal Models

Preclinical research that focused on the role of ECS in schizophrenia relies on the evaluation of sensorimotor gating deficits by the prepulse inhibition (PPI) paradigm (245, 246). Among all the components of the ECS, CB1r is critically involved in schizophrenia. In fact, results of studies using pharmacological approaches showed that CB1r activation induces psychotic-like effects, while blockade of CB1r presents opposite actions. Decreases in startle responses together with PPI disruption were achieved by CP-55,940 administration, and rimonabant completely reversed these effects (247). A similar experiment was carried out in which CP-55,940 decreased startle response and impaired PPI, and rimonabant significantly reversed CP-55,940-induced deficits in PPI only at the lower prepulse intensity (248). This CB1r-mediated auditory gating disruption was further confirmed by measuring neuronal network oscillations in the Hipp and entorhinal cortex of Sprague-Dawley rats. CP-55,940 significantly impaired sensory gating and neuronal oscillation, an effect that was reversed by AM251 (249).

After learning that the modulation of the CB1r produced sensorimotor alterations, different animal models of schizophrenia were used to find out if CB1r blockade could be a strategy with therapeutic potential. Blockade of N-methyl-D-aspartate (NMDA) receptors (NMDAr) was used to simulate schizophrenia-like symptoms in rodents (250). Interestingly, the administration of AM251 significantly abolished phencyclidine-induced disruption of PPI in a similar way to clozapine (251), as well as impairments in recognition memory or increased behavioral despair in the FST (252). Another NMDAr antagonist used to model schizophrenia-like behavior is MK-801. The administration of the CB1r antagonist AVE1625 reversed MK801-induced cognitive impairments and decreased catalepsy and weight gain induced by clinically used antipsychotic drugs (haloperidol, olanzapine) (253). Furthermore, AM251 attenuated amnesic effects and hyperactivity induced by MK-801 (254). Therefore, it appears that blockade of CB1r may have relevant therapeutic applications for the treatment of schizophrenia.

In the so-called ‘three-hit' animal model of schizophrenia, CB1r binding and cannabinoid agonist-mediated G-protein activation decreases in the cortical, subcortical, and cerebellar brain regions (255). In a neurodevelopmental animal model of schizophrenia induced by the gestational administration of methylazoxymethanol (MAM), CB1r mRNA levels were lower in the PFC and higher in the dorsolateral striatum of adult MAM-treated Sprague-Dawley rats relative to the control group (256). Moreover, in the spontaneously hypertensive rat (SHR) strain, partially reproducing some schizophrenia-like behavioral aspects, CB1r immunoreactivity was significantly increased in the PL, cingulate cortex, and CA3 region of the Hipp (257). Recently, two studies found decreased methylation of the cannabinoid receptor interacting protein (CNR1P1) DNA promoter in the ventral Hipp (vHipp) of rats exposed to the MAM model (258, 259). CNR1P1 is an intracellular protein that interacts with the C-terminal tail of CB1r and regulates its intrinsic activity. Interestingly, a lentivirus-mediated overexpression of CNR1P1 in the vHipp of Sprague-Dawley rats induced significant schizophrenia-like cognitive and social interaction impairments, together with an increase of dopamine neuron population activity in the VTA (260).

Apart from the many preclinical studies supporting the pivotal role of CB1r, animal models of schizophrenia provided interesting results about eCBs brain level alterations. In this regard, a significant increase in 2-AG levels in the PFC of PCP-treated Lister-Hooded rats was reversed by treatment with THC, which in turn induces a large reduction of AEA in the same region (261). Furthermore, in Sprague-Dawley rats exposed to a bilateral olfactory bulbectomy, considered as an animal model of depression and schizophrenia, a significant decrease of AEA and 2-AG levels was found in the ventral striatum (262). In addition, mice with a heterozygous deletion of neuregulin 1 (Nrg 1 HET mice), a well-accepted and characterized animal model of schizophrenia (263), displayed relevant alterations in eCBs levels (264).

Finally, CB2r has also been recently involved in schizophrenia. Ishiguro and cols. studied the effects of the pharmacological blockade of CB2r in two animal models of schizophrenia induced by the administration of MK-801 or metamphetamine. The CB2r antagonist AM630 significantly exacerbated the MK-801- or metamphetamine-induced hyperlocomotion and PPI disruption, suggesting that CB2r was mediating these actions (265). Our group analyzed exhaustively the behavioral profile of CB2^−/−^ mice to evaluate the implication of CB2r in schizophrenia-like behavior. The phenotype showed by CB2^−/−^ mice resembled some relevant features of schizophrenia such as increased sensitivity to motor effects of cocaine, anxiety- and depressive-like behavior, disrupted short- and long-term memory consolidation and impaired PPI. These behavioral alterations were accompanied by gene expression changes in different targets from dopaminergic, noradrenergic, and serotonergic systems. Interestingly, the atypical antipsychotic risperidone significantly improved PPI disruption induced by CB2r deletion and differentially modulated some of the neurochemical disturbances compared with WT mice (266). In addition, the activation of CB2r by the agonist JWH015 reversed PPI disruptions of the MK-801-induced animal model of schizophrenia, and this effect was specifically mediated by CB2r since only AM630 but not AM251 abolished PPI improvement (267). Furthermore, activation of CB2r (JWH133) and blockade (AM630) increased MK-801-induced hyperlocomotion, although this effect was much more evident and pronounced with AM630 (268). Therefore, these results strongly suggest that CB2r functional regulation is significantly involved in schizophrenia-like behavior.

#### Human Studies

To date, an extensive and great effort has been made to elucidate the role that CB1r plays in schizophrenia. Accumulated clinical data clearly shows significant alterations of CB1r protein and gene expression levels, as well as certain CNR1 polymorphisms correlations, especially in the brain but also in the peripheral blood cells from schizophrenic patients in comparison with healthy control subjects. The information reviewed and detailed below provides important clues to further investigate the application of CB1r-related measures as potential trait, state, prognostic or even therapeutic biomarkers.

Several published studies analyzed CB1r protein and gene expression levels in different *post-mortem* brain regions from schizophrenic patients. Several studies examined quantitative autoradiography to evaluate CB1r availability through the binding of different radioligands. A significant increase in CB1r availability was shown in the dorsolateral prefrontal cortex (DLPFC) (269–271), although this increase was only present in paranoid schizophrenic patients (272). Interestingly, a recent study failed to show differences in CB1r-mediated functional coupling to G-proteins in the PFC of schizophrenic and control patients (273). Furthermore, higher CB1r binding levels were shown in the left ACC (274) and in superficial layers of the posterior cingulate cortex (PCC) (275), whereas no changes were found in the superior temporal gyrus (STG) (276) from schizophrenic patients. In contrast, some authors reported lower CB1r protein levels measured by immunocytochemistry (277) or Western blot (278) and decreased CB1r gene expression analyzed by *in situ* hybridization (277) or quantitative real time polymerase chain reaction (qRT-PCR) (273) in the PFC from schizophrenic patients compared with control subjects. Volk and cols. specifically addressed this apparent discrepancy between CB1r binding and protein or gene expression levels. In a cohort of 21 schizophrenic patients presenting lower levels of both CB1r mRNA and protein in the PFC, relative to matched healthy comparison subjects, they obtained an increased CB1r binding (271).

Neuroimaging experiments were recently carried out to obtain an *in vivo* approximation of the disturbances related with CB1r in schizophrenia. In this regard, PET studies yielded dissimilar results. Wong and cols. studied CB1r binding employing the novel PET tracer [^11^C]-OMAR (JHU 75528) in schizophrenic patients and matched controls. CB1r binding was higher in several brain regions of patients with schizophrenia, only reaching statistical significance in the pons. Interestingly, a significant correlation was found between CB1r binding and schizophrenia-related symptomatology (279). In addition, Ceccarini and cols. also showed a significant increase of CB1r binding in the NAc, insula, cingulate cortex, inferior frontal cortex, parietal and mediotemporal lobes in schizophrenic patients compared with controls measured with [^18^F]-MK-9470 PET. It is relevant to highlight that in the nontreated schizophrenia patients, CB1r binding was negatively correlated to negative symptoms and to depression scores, especially in the NAc (280). On the contrary, Rangathan and cols. obtained an opposite result with lower CB1r availability levels ([^11^C]-OMAR PET) in the Amy, caudate, PCC, Hipp, Hyp, and insula of schizophrenic patients (281). An interesting commentary on these discrepancies was published, in which several confounding factors such as symptom severity, sex, age, PET tracer, statistical analysis method or comorbid nicotine use are discussed. Overall, it could be concluded that CB1r has an important but yet complex and poorly understood role in schizophrenia (282). Finally, a very recent study examined CB1r availability by [^18^F]-FMPEP-d2 or [^11^C]-MePPEP PET, in first episode psychosis (FEP). Significant lower CB1r availability was found in patients with schizophrenia, independently of antipsychotic medication treatment. Greater reduction in CB1r availability was associated with greater symptom severity and poorer cognitive functioning (283).

The possible association between CNR1 polymorphisms and schizophrenia has been explored. In this sense, negative results were obtained with a single-base polymorphism within the first exon of the CNR1 (284), the polymorphism rs1049353 1359G/A at codon 453 in the coding region of CNR1 (285–288), or other CNR1 polymorphisms such as rs6454674 (287), AL136096 (287), rs806368 (288, 289), rs806379 (288), rs806380 (288), rs806376 (289), and rs806366 (289). However, significant associations of CNR1 polymorphisms rs7766029, rs806366, and rs1049353 were described (290). Regarding (AAT)n triplet repeat in the promoter region of the CNR1 gene, discrepant results were reported since Tsai and cols. suggested that this polymorphism was not directly involved in the pathogenesis of schizophrenia in a Chinese population (291), whereas it was significantly associated with the hebephrenic or disorganized subtype of schizophrenia (285). Interestingly, some relevant associations were identified between specific CNR1 polymorphisms and therapeutic response. Hamdani and cols. described increase G-allele frequency of the rs1049353 polymorphism in responsive schizophrenic patients, with a dose effect of the G allele. Thus, the authors proposed that the G allele of CNR1 rs1049353 polymorphism could represent a “psychopharmacogenetic” biomarker to take into consideration for the treatment of schizophrenia (288). In addition, in 65 FEP patients, TT genotype of the CNR1 rs2023239 polymorphism was associated with a better improvement of negative and positive symptoms (292). Similarly, in another group of patients with FEP, carriers of rs7766029 CC genotype presented significantly higher improvement in verbal memory and attention while carriers of rs12720071 AG genotype showed a better improvement in executive functions (293). Furthermore, minor alleles of CNR1 polymorphisms rs6928499, rs1535255, and rs2023239 might be associated with a lower risk to develop antipsychotic-induced metabolic syndrome. These relevant data could result in potential pharmacogenetic applications to optimize drug management of schizophrenic patients (294). On the contrary, one study showed that G allele carriers of the CNR1 rs1049353 (G1359A) polymorphism might be associated with a poorer therapeutic response (233).

Gene and protein analysis of CB1r in peripheral blood cells from schizophrenic patients attracted much attention. Peripheral cell (*e.g.* lymphocytes) changes could be mirroring, at least in part, some of the neuropathological hallmarks of the disorder. In this regard, the first published study did not detect changes in the CB1r mRNA levels in peripheral blood mononuclear cells (PBMCs) between schizophrenia and control patients (295). Similarly, no differences were observed in CB1r levels of peripheral immune cells by flow cytometry between control and schizophrenic patients, although a positive correlation between CB1r expression on monocytes and cognitive impairment was detected (296). However, an opposite result revealed an increase of CB1r in PBMCs of schizophrenic patients also evaluated by flow cytometry (297). Furthermore, there is an increase of CB1r mRNA levels in PBMCs of schizophrenic patients (298, 299) that may correlate with a reduced DNA methylation of CNR1 promoter region (299). Moreover, CB1r gene expression was correlated positively with positive and negative syndrome scale (PANSS) total symptom severity and negatively with cognitive functioning measures (298).

Besides the extensive literature evaluating the role of CB1r in schizophrenia, some efforts were done to complete the picture regarding the involvement of eCBs and its degrading and synthesizing enzymes as biomarkers. Leweke and cols reported a significant increase of AEA levels in the CSF of schizophrenic patients (300). In antipsychotic naïve first-episode paranoid schizophrenic patients, there was an eightfold increase in AEA levels in the CSF, whereas no alteration was present in patients treated with typical but not atypical antipsychotics. Furthermore, AEA levels were negatively correlated with psychotic symptoms in nonmedicated acute schizophrenics (301). Similarly, blood AEA levels were higher in patients with acute schizophrenia and were normalized with the clinical remission (295). Increased AEA levels were also detected in the CSF of schizophrenic patients who used cannabis. Interestingly, the increase of AEA was more than 10-fold higher in low-frequency compared with high-frequency cannabis users (302). In addition, higher AEA serum levels were obtained in twin-pairs discordant for schizophrenia (303), or in schizophrenic patients with substance use disorder (SUD) comorbidity, considering that baseline AEA predicted endpoint SUD scores (304). However, other studies showed different results such as no changes in serum AEA levels (305), increased 2-AG and decreased AEA in the cerebellum, Hipp, and PFC of schizophrenic patients (306).

With regard to degrading or synthesizing eCBs enzymes, the relationship of some FAAH or NAPDE-PLD polymorphisms with schizophrenia was studied, but no significant associations were obtained (290, 307). In addition, FAAH and MAGL mRNA levels were similar while FAAH activity was higher in the PFC of schizophrenic patients compared to controls (273). Interestingly, a reduction of FAAH mRNA levels correlated with clinical remission in schizophrenic patients (295). Furthermore, in FEP patients, some interesting correlations were detected between peripheral FAAH and DAGL expression and short-term verbal memory, NAPE-PLD expression and working memory, and MAGL expression and attention. Accordingly, the authors suggested the use of these ECS elements as biomarkers or pharmacological targets for FEP (308). Finally, mRNA levels of the 2-AG metabolizing enzyme, *α*-*β*-hydrolase domain 6 (ABHD6), were significantly increased in patients with schizophrenia (309).

Notwithstanding the scarce literature exploring the role of CB2r in schizophrenia, some important findings suggest its involvement and draw attention to research on its therapeutic potential. Perhaps, de Marchi and cols. published the first evidence measuring CB2r mRNA levels by semi-quantitative RT-PCR in PBMCs from schizophrenic patients in their acute phase, and when clinical remission was achieved after antipsychotic treatment with olanzapine. CB2r gene expression significantly decreased in PBMCs from patients in clinical remission (295). In FEP patients, CB2r protein expression was significantly down-regulated together with reduced levels of eCBs synthesizing enzymes (NAPE-PLD and DAGL) (310). Interestingly, increased CB2r gene expression was found in schizophrenic patients' PBMCs (298), correlating with PANSS and cognitive performance severity (296, 298), and in cells of the innate immune system (297). On the other hand, Ishiguro and cols. evaluated the implication of specific CNR2 polymorphisms in schizophrenia. R63 allele of rs2501432 (R63Q), C allele of rs12744386, and the haplotype of the R63-C allele were significantly increased in patients with schizophrenia in comparison with control subjects. Apparently, these polymorphic alterations of CNR2 are associated with loss of function. A lower response to CB2r ligands was found in cultured CHO cells transfected with the R63 allele. Reduced CB2r mRNA and protein expression levels were found in the DLPFC of schizophrenic patients independently of the diagnosis (265). In addition, the association between three CNR2 polymorphisms (rs2501432C/T, rs2229579C/T, rs2501401G/A), and schizophrenia was explored (311). However, other CNR2 polymorphisms (rs6689530 and rs34570472) were not associated with schizophrenia in a Korean population (289).

### Bipolar Disorder

Bipolar disorder (BD) is a debilitating, lifelong neuropsychiatric illness characterized by unsteady mood states alternating from (hypo)mania to depression. According to the DSM-5, for a diagnosis of BD it is necessary to meet specific criteria for a manic episode that may be followed by hypomanic or major depressive episodes (63). Despite the availability of effective pharmacological agents, BD is inadequately treated in a subset of patients, so the identification of new therapeutic targets is necessary. In this sense, the close implication of ECS in mood regulation suggested its involvement in BD (312). This assumption is supported by the observation of the effects of high doses of cannabis and THC in healthy patients, producing psychosis, sometimes with marked hypomanic features (313). In addition, THC and cannabidiol (CBD), the main components of *Cannabis sativa* plant, may present mood stabilizing properties. Therefore, there is an increasing interest to evaluate ECS implication in BD.

First studies were focused on the evaluation of polymorphisms of CNR1 gene in BD pathophysiology. One study carried out in patients with BD within a Turkish population investigated the implication of three types of polymorphisms of CNR1 in this disease, demonstrating that only one of them (rs6454674) could be correlated with BD. In addition, the mean of the yearly maniac attacks was statistically higher in patients presenting heterozygote rs6454674 T/G polymorphisms compared to those with homozygote polymorphism (314). In addition, the association of CNR1 rs1049353 (1359 G/A) and FAAH rs324420 SNP (cDNA 385C to A) polymorphisms with BD was assessed in a Caucasian population. Here, the authors concluded that the distribution of CNR1 1359 G/A genotypes and alleles did not differ between BD and healthy patients, whereas the frequency of the AC genotype of FAAH (cDNA 385C to A) polymorphism was slightly higher in BD patients (232).

Nevertheless, other studies did not identify differences in ECS components between BD and healthy controls. Indeed, no differences were obtained between BD patients and healthy controls in DNA methylation of the CNR1 gene promotor region (299). Furthermore, a polymorphism of CNR1 promotor region was evaluated in another study, and no changes were observed in BD patients, concluding that it was not likely to relate with BD (315). Koethe and cols. carried out a study with *post-mortem* brain samples from BD patients and controls, evaluating numerical density of neurons and immunopositive glial cells for CB1r. No changes were found in these patients (223). Furthermore, in another study evaluating polymorphisms of CNR1 and FAAH, no significant differences or association were observed in BD patients (316).

Because of these contradictory results, some authors shifted their attention to the implication of CB2r in BD, with limited but promising findings. A genetic association was observed in patients with BD and CNR2 rs41311993 (524C/A) polymorphism, but not SNPs of rs2229572 (1073C/T) or rs2501432 (315A/G), suggesting that CB2r may play a role in BD (317). In addition, a genome-wide association study carried out in a population from the UK biobank, identified the association of a locus in CNR2 with distressing psychotic experiences, providing support for a shared genetic liability with BD and other neuropsychiatric disorders (318).

In summary, there is limited information about the implication of ECS in the pathophysiology of BD. Thus, more preclinical and clinical studies are needed to explore further its role in the development of this neuropsychiatric disorder and its usefulness as a therapeutic target to improve BD management.

### Post-Traumatic Stress Disorder

Post-traumatic stress disorder (PTSD) is a chronic and disabling mental disease caused by the exposure to stressful, frightening or distressing events, and is included in the category of trauma- and stressor-related disorders in the DSM-5 (63). PTSD patients experience intrusion symptoms, persistent avoidance of any stimuli associated with the traumatic event, negative alterations in cognition and mood, and disturbances in arousal and reactivity, that must last more than 1 month and produce distress or functional impairment (63). The neurobiological mechanisms underlying PTSD-related symptomatology are not completely understood, being a limiting factor to identify new therapeutic targets. In this regard, a relevant association between ECS and PTSD was suggested providing interesting results about the potential development of new pharmacological approaches. Indeed, preclinical and clinical findings point out the involvement of certain ECS components in PTSD symptomatology, such as CB1r or FAAH, suggesting its potential role as biomarkers for PTSD (319, 320).

#### Animal Studies

The involvement of CB1r in PTSD is supported by the presence of this receptor in brain areas regulating the response to stress and to changes observed in different animal models of PTSD. For instance, using a shock and reminder model of PTSD, higher mRNA levels of CB1r were detected in the BLA (133), and increased CB1r protein expression was found in the BLA and the CA1 region of the Hipp (321) of exposed mice as well. On the other hand, in a predator exposure-based PTSD model, anxiety-like behavior was negatively correlated with CB1r gene expression in the PFC and the amygdaloid complex, whereas no changes were observed in the Hipp (322). In addition, Xing and cols. reported in young Sprague-Dawley rats, that the exposure to an unpredictable electric shock model of PTSD induced a down-regulation of CB1r gene expression in comparison with nonstressed rats. Interestingly, the authors showed sex differences in the stress-related regulation of CB1r, showing that females presented higher mRNA levels of CB1r, as well as greater CB1r inactivation by phosphorylation. The authors concluded that these sex-related differences could lead to increased susceptibility to stress-related anxiety disorders, including PTSD, in females (323). A genetic approach was used to evaluate further the involvement of CB1r in the regulation of stress response. Repeated exposure to an acoustic stressor (high intensity bell sound) did not produce changes in adrenocorticotropin hormone (ACTH) or corticosterone (CS) levels in CB1^−/−^ mice. These results suggested that the presence of CB1r is essential in the regulation of the stress response, and that CB1^−/−^ mice may result appropriate to model some forms of PTSD (324).

Pharmacological manipulation approaches of the CB1r were also explored in several rodent models of PTSD and its potential usefulness as a therapeutic biomarker. The blockade of CB1r with rimonabant increased freezing behavior in a PTSD model of shock and reminder during cued expression/extinction training (325). On the other hand, several authors evaluated the effects of cannabinoid activation by WIN-55,212-2 administration into hippocampal CA1 region. The results showed a normalization of shock-induced upregulation of CB1r in the PFC and CA1 region of the Hipp (321, 326) and facilitation of inhibitory avoidance extinction in a fear-related inhibitory avoidance paradigm (327). All these effects were blocked by AM251 administration. Furthermore, WIN-55,212 administration into the BLA normalized stress-induced effects on inhibitory avoidance and acoustic startle response and facilitated fear extinction in a single prolonged stress (SPS) model of PTSD. These effects were blocked by AM251 (321, 328, 329). Similarly, the injection of WIN-55,212 in the NAc of rats exposed to a shock and reminder model of PTSD significantly facilitated the fear extinction process (330). Interestingly, Goodman and Packard demonstrated that systemic or intradorsolateral striatum (DLS) administration of WIN-55,212 could impair the consolidation of stimulus–response memory, suggesting relevant consequences for neuropsychiatric disorders such as PTSD (331). However, the intra-PFC administration of WIN-55,212 did not modulate fear extinction disturbances induced by the exposure to the SPS model (329). Finally, according to the results obtained with WIN-55,212, Reich and cols. studied the effects of a CB1r selective agonist, ACEA, in rats exposed to 3 weeks of a chronic-mild-unpredictable protocol followed by fear conditioning evaluation. In this study, ACEA administration significantly reduced freezing behavior in stressed rats, enhancing long-term extinction of fear-related memories (332).

In order to validate that cannabinoid activation improves disturbances induced by stress- or trauma-related stimuli, the effects of pharmacological endocannabinoid signaling facilitation were analyzed. In a fear conditioning paradigm, the administration of AM404 led to a dose-dependent enhancement in fear extinction, as well as a decreased shock-induced reinstatement of fear. Interestingly, the administration of rimonabant together with AM404 reversed the improvement of extinction, suggesting that AM404 effects were related to an increase in CB1r activation during extinction training (333). In addition, the injection of the FAAH inhibitor, URB597, normalized the upregulation of CB1r in the CA1 of Hipp and BLA of rats exposed to a shock and reminder model of PTSD (334) and attenuated startle response and anxiety-like behavior in a predator exposure animal model of PTSD (335). Interestingly, these effects were abolished by CB1r blockade, suggesting the implication of CB1r on URB597 effects (335, 335). Similar results were obtained with the administration of URB597 into CA1 (Hipp) and BLA brain regions, showing a facilitation of extinction processes and attenuation of startle response, anxiety- and depression-like behaviors mediated by CB1r activation (133, 336, 337). Furthermore, URB597 administration additionally prevented the increase of CB1r levels in CA1 and BLA after rodent exposure to shock and reminder model of PTSD (321).

FAAH inhibition significantly facilitates CB1r-mediated signaling of AEA and can produce a greater beneficial spectrum of biological effects than those caused by direct CB1r activation. Interestingly, the role of FAAH in learning and memory was evaluated by using another FAAH inhibitor, OL-135. The administration of this drug increased acquisition and extinction rates in mice exposed to fixed platform water maze test. Rimonabant blocked OL-135-induced effects on both acquisition and extinction levels (338). In the same study, the authors revealed that FAAH^−/−^ mice phenotype was similar to that obtained after OL-135 administration, suggesting that the increase in AEA levels facilitates extinction processes, and that CB1r would be critically involved (338). FAAH inhibition and the consequent increase of AEA in the brain regions involved in the regulation of stress and anxiety seem to restore dysfunctional homeostasis of AEA signaling because of stress exposure. Thus, FAAH must be strongly considered as a target for PTSD management (139).

#### Human Studies

According to the involvement of ECS components in several behavioral traits of PTSD in animal models, various studies explored alterations in different biological samples (*post-mortem* brain tissue, blood, hair) collected from PTSD patients and adequately paired controls. At the peripheral level, some authors studied the possible correlation between CNR1 polymorphisms and PTSD symptoms. The rs1049353 polymorphism of CNR1 was studied in PTSD patients to correlate specific alleles or genotypes with fear and/or dysphoric symptoms of PTSD. This study suggested that rs1049353 polymorphism interacts with childhood physical abuse to increase fear but not dysphoric symptoms in PTSD (339). In another study carried out in a Caucasian population, the association between variants of CNR1 gene haplotypes and diagnosis of PTSD was studied. The authors reported that the variant C-A was more common in PTSD cases compared to non-PTSD controls, and the variant C-G was less common in PTSD compared to non-PTSD patients (340).

A different approach was the measurement of plasmatic eCBs in a selected cohort of patients that suffer the terroristic attacks of the World Trade Center in 2001 and met the diagnostic criteria for PTSD. 2-AG, AEA, and cortisol concentrations were measured. Only 2-AG was significantly reduced in PTSD patients, while no significant differences were found in AEA or cortisol concentrations (341). Another study showed reduced AEA and cortisol concentrations in PTSD patients compared to healthy controls with lifetime clinical histories of trauma (342). Despite these contradictory results, the fluctuations in plasmatic concentrations of eCBs may affect the reproducibility of the evaluation. Thus, the assessment of eCBs alterations in hair samples provides a more stable measurement. Hair concentrations of PEA (palmitoylethanolamide), OEA (oleoylethanolamide) and SEA (steraoylethanolamide) were measured in war survivors with and without PTSD. A regression analysis revealed a strong negative relationship between these endocannabinoids and the severity of PTSD symptoms. OEA concentrations were significantly reduced in hair samples from PTSD patients (343).

Only one human study analyzed CB1r binding using the CB1r-selective radioligand [11C]OMAR by PET. Results showed elevated CB1r binding values, especially in women, together with lower AEA and cortisol in PTSD patients. The authors suggested that abnormal CB1r-mediated AEA signaling is involved in the etiology of PTSD (342). In addition, fMRI was also used to evaluate FAAH implication in PTSD symptomatology. A common SNP (C385A) in the human FAAH gene was correlated with the quicker habituation of amygdala reactivity to threat and lower score on stress-reactivity. This variant reduced FAAH activity and possibly increased AEA-induced endocannabinoid signaling (344–346). Furthermore, Rabinak and cols. conducted an fMRI study with healthy volunteers and patients receiving acute dronabinol (synthetic THC) oral administration in a standard Pavlovian fear extinction paradigm. Interestingly, dronabinol enhanced extinction learning, providing the first evidence about the feasibility of pharmacological enhancement of extinction learning in humans using cannabinoid system modulators (347, 348). Some clinical trials with PTSD patients suggested the usefulness of dronabinol for improving the global PTSD symptom severity, sleep quality, frequency of nightmares, and PTSD hyperarousal symptoms (349). Similar results were obtained with nabilone, since its administration to PTSD patients improved insomnia, PTSD symptoms, and global assessment of functioning, reducing the frequency and intensity of nightmares (350, 351). Nevertheless, more randomized and controlled clinical trials are needed to confirm dronabinol or nabilone potential therapeutic application in the management of PTSD.

### Attention-Deficit/Hyperactivity Disorder

ADHD is a neuropsychiatric disorder characterized by persistent pattern of inattention and/or hyperactivity-impulsivity that interferes or reduces the quality of social, academic, or occupational functioning in accordance with DSM-5 (63). In the last years, the identification of different components of the ECS that are potentially involved in ADHD pathophysiological mechanisms has attracted much attention as shown below.

#### Animal Studies

An experiment carried out in SHR rats (an animal model reproducing some features of ADHD) evaluated the modulating effects of the cannabinoid system on impulsivity, using a delay reinforcement task and the administration of WIN55212-2 or AM251 (352). This study concluded that treatment with WIN55212-2 decreased whereas AM251 increased the choices of the large reward, suggesting that CB1r plays a relevant role in impulsive behavior. Furthermore, basal gene and protein expression of CB1r in the brainstem of SHR rats was significantly lower in comparison with their normotensive counterpart, Wistar rats (353). Moreover, the overexpression of four genes, between them CNR1, was strongly associated with overall poor performance on mice during their gestational growth because of a malnutrition *via* high-fat or low-protein diets on the dam (354). These abnormal disturbances on diet in the gestational period are linked to the etiology of multiple neurodevelopmental disorders, including ADHD (355).

The psychostimulant drug amphetamine is often prescribed to treat ADHD. The administration of amphetamine increases monoamine neurotransmission in the brain regions as NAc and medial PFC. Accumulating reports supported the role of CB1r in the regulation of monoamine release, suggesting its possible involvement in ADHD. The administration of rimonabant did not affect monoamine release whereas dose-dependently abolished amphetamine-induced dopamine release in the NAc. This result suggested that CB1r is essential to reach the therapeutic effect of amphetamine, mediated at least in part, by the enhancement of dopaminergic signaling in the mesolimbic system in the NAc (356).

#### Human Studies

As previously stated, there is large available evidence regarding the role of different variants of CNR1 gene in psychiatry. In relation to ADHD, SNP variants at the CNR1 gene were tested on a family-based sample of trios (an ADHD child and their parents) and on an unselected adolescent sample from Northern Finland. The study detected a significant association of a SNP haplotype (C-G) with ADHD suggesting a greater risk in males than females (340). Another study reported the interaction of the two most studied *CNR1* polymorphisms, rs806379 and rs1049353, that are involved with early psychosocial adversity (357). These polymorphisms of the CB1 receptor are highly associated with impulsivity representing an usual phenotype involved in ADHD (358).

### Eating Disorders

The most common eating disorders are anorexia nervosa (AN) and bulimia nervosa (BN). According to DSM-5, AN is characterized by distorted body image and excessive dieting leading to severe weight loss with a pathological fear of becoming fat, whereas BN is characterized by recurrent episodes of binge eating alternated with recurrent inappropriate compensatory behavior to prevent weight gain (63). Importantly, ECS plays a major regulatory role on feeding behaviors and energy balance (359) that has drawn attention to its relationship with ADHD neurobiology. This can be confirmed by rodents with diet-induced or genetic obesity promoting an increase of endocannabinoid hypothalamic levels (360).

#### Animal Studies

The participation of CB1r in the regulation of feeding behavior is well established. The acute administration of rimonabant decreased food intake and body weight gain and reduced CB1r gene expression in the PVN of male Wistar rats. However, a chronic treatment led to tolerance to the hypophagic effects of CB1r blockade without changes in food intake, body weight, or hypothalamic mRNA gene expression (361). Another study on rimonabant-treated male Sprague-Dawley rats showed that the colocalized Fos labeling of hypothalamic regions with anorexigenic and orexigenic peptides had decreased neuropeptide Y (NPY) levels (362). More recently, another study was performed in CB1r conditional and CB2^−/−^ mice. The hypothalamic neuropeptide expression pattern displayed a marked decrease of proopiomelanocortin (POMC) and cocaine-amphetamine-regulated transcript (CART) expression in the arcuate nucleus of the hypothalamus (ARC), both neuropeptides involved on anorexigenic and behavioral changes in food intake (363).

C57BL/6J mice were treated with naltrexone (opioid receptor antagonist), rimonabant, and BD-1063 (sigma-1 receptor antagonist) on an intermittent maladaptive feeding animal model. All the treatments reduced overconsumption of a palatable food (364). In addition, the administration of other cannabinoid compounds such as CBD or CB1r antagonists significantly reduced food intake and body weight gain (365–368). Recently, a study based on the activity-based anorexia (ABA) model reproducing key aspects of human AN, measured levels of AEA, 2-AG, and the CB1r in different brain regions of female ABA Sprague-Dawley rats. 2-AG significantly decreased in various brain areas but not in the caudate putamen, whereas no changes were observed in AEA. Density of CB1r was reduced in the dentate gyrus of Hipp and in the lateral Hyp (369). These results suggested that ECS is involved in the contribution and maintenance of some aspects in the pathophysiology of AN.

#### Human Studies

Although there is some progress in the understanding of the mechanisms underlying eating disorders and body weight regulation, there is still lack of information to suggest cannabinoid related treatment for patients with AN and BN. However, some studies suggest that the ECS, primarily CB1r, plays a key role in the reward areas and metabolic patterns involved in food intake and weight gain. In this regard, a PET study on 54 patients with food intake disorders (FID, including AN and BN) revealed an inverse association between regional CB1r availability and body mass index (BMI) in the Hyp and brainstem areas in both patients with FID and healthy individuals. However, FID patients negatively correlated with BMI throughout the mesolimbic reward system (370). Also, global CB1r availability is significantly increased in the cortical and subcortical brain areas in AN patients compared with healthy controls, maybe due to a compensatory mechanism of an underactive ECS in these patients (371). Finally, eating disorder female patients presented lower CB1r mRNA levels in PBMCs (372).

## Conclusions

The close involvement of the ECS in the etiology and neuropathology of neuropsychiatric disorders is undeniable. Considering the urgent need to identify new and better biomarkers in psychiatry, the evidence included in this review provides an overview of the opportunities that cannabinoid receptors (**Table 1**), endogenous cannabinoid ligands (**Table 2**), or their metabolizing enzymes (**Table 3**) offer as potential biomarkers in the clinical setting. The large number of pharmacological studies with various cannabinoid compounds, mainly conducted in animal models, reported interesting and promising information to design new therapeutic strategies that, alone or in combination with the drugs currently used in psychiatry, may improve the efficacy and safety of psychiatric disorders treatment.

**Table 1:** Main findings from human studies supporting the role of CB1r and CB2r as biomarkers in psychiatric disorders.

CB1r					
Subjects/Diagnosis	Sample/Intervention	Method	Measurement	Results	References
Healthy controls	THC (10 mg) p.o.	[^11^C]MePPEP PET	CB1r availability in amygdala	↑ CB1r	Bhattacharyya et al. (166)
Healthy controls	Rimonabant (90 mg) p.o.	Visual Analogue Mood Scale	Anxiety level	↑ anxiety	Bergamaschi et al. (168)
AD/DD	Buccal mucosa cells	DNA Genotyping	CNR1 rs7766029 polymorphism	↑ frequency financial-related anxiety and depression	Gonda et al. (170)
DD	PMBT – anterior cingulate cortex	Immunohistochemistry	Density of CB1r immunopositive glial cells	↓ CB1r	Koethe et al. (223)
DD	PMBT – dorsolateral prefrontal cortex	Quantitative polymerase chain reaction (qPCR)	CB1r relative gene expression	↑ CB1r	Choi et al. (224)
DD	Blood	DNA Genotyping	CNR1 rs1049353 (1359 G/A) polymorphism	↑ frequency	Monteleone et al. (232)
DD/SCZ	Blood	DNA Genotyping	CNR1 rs1049353 (1359 G/A) polymorphism	↑ depressive symptoms in G-allele carriers	Schennach et al. (233)
DD	Blood	DNA Genotyping	CNR1 rs1049353 (1359 G/A) polymorphism	↑ treatment resistance in G-allele carriers	Domschke et al. (234)
DD	Blood	DNA Genotyping	CNR1 rs1049353 (1359 G/A) polymorphism	↑ citalopram response in GG genotype male carriers	Mitjans et al. (235)
Missouri Adolescent Female Twin Study (MOAFTS) participants	Blood	DNA Genotyping	CNR1 rs1049353 (1359 G/A) polymorphism	↓ risk for anhedonia/DD in A-allele carriers with childhood trauma	Agrawal et al. (236)
DD in Parkinson's disease	Blood	DNA Genotyping	CNR1 (AAT)n triplet polymorphism	↓ risk for DD in 2 long alleles carriers	Barrero et al. (238)
DD in methadone-maintained patients	Blood	DNA Genotyping	CNR1 rs2023239 polymorphism	↓ risk for DD in C-allele carriers	Icick et al. (239)
SCZ	PMBT – dorsolateral prefrontal cortex	In situ [^3^H]CP-55940 radioligand binding	CB1r binding	↑ CB1r	Dean et al. (269)
SCZ	PMBT – dorsolateral prefrontal cortex	In situ [^3^H]MePPEP radioligand binding	CB1r binding	↑ CB1r	Jenko et al. (270)
SCZ	PMBT – prefrontal cortex	In situ [^3^H]OMAR radioligand binding	CB1r binding	↑ CB1r	Volk et al. (271)
SCZ	PMBT – anterior cingulate cortex	In situ [^3^H]SR141716A radioligand binding	CB1r binding	↑ CB1r	Zavitsanou et al. (274)
SCZ	PMBT – posterior cingulate cortex	In situ [^3^H]CP-55,940 radioligand binding	CB1r binding	↑ CB1r	Newell et al. (275)
SCZ	PMBT – posterior cingulate cortex	In situ hybridization Immunohistochemistry	CB1r mRNA CB1r protein	↓ CB1r ↓ CB1r	Eggan et al. (277)
SCZ	PMBT – prefrontal cortex	Western Blot	CB1r protein	↓ CB1r ↓ CB1r in antipsychotic-treated patients	Urigüen et al. (278)
SCZ	PMBT – prefrontal cortex	Quantitative polymerase chain reaction (qPCR)	CB1r relative gene expression	↓ CB1r	Muguruzaf et al. (273)
SCZ	*In vivo* neuroimaging (several brain regions)	[^11^C]-OMAR PET	CB1r binding	↑ CB1r (only in the pons)	Wong et al. (279)
SCZ	*In vivo* neuroimaging (several brain regions)	[^18^F]-MK-9470 PET	CB1r binding	↑ CB1r	Ceccarini et al. (280)
SCZ	*In vivo* neuroimaging (several brain regions)	[^11^C]-OMAR PET	CB1r binding	↓ CB1r	Ranganathan et al. (281)
FEP	*In vivo* neuroimaging (several brain regions)	[^18^F]-FMPEP-d2 or [^11^C]-MePPEP PET	CB1r binding	↓ CB1r ↔ severity	Borgan et al. (283)
SCZ	Blood	DNA genotyping	CNR1 rs1049353, rs7766029, rs806366 polymorphisms	Nominal association	Costa et al. (290)
SCZ	Blood	DNA genotyping	CNR1 (AAT)n triplet polymorphism	9 and 17 repeat alleles ↔ ↑ susceptibility disorganized SCZ	Ujike et al. (285)
SCZ	Blood	DNA genotyping	CNR1 rs1049353 (1359 G/A) polymorphism	↑ treatment response in G-allele carriers	Hamdani et al. (290)
SCZ	Blood	DNA genotyping	CNR1 rs2023239 polymorphism	↑ better improvement in TT genotype carriers	Suárez-Pinilla et al. (292)
FEP	Blood	DNA genotyping	CNR1 rs7766029, rs12720071 polymorphisms	↑ better improvement in rs7766029 CC genotype or rs12720071 AG genotype	Kuzman, R. et al. (293)
SCZ	Blood	DNA genotyping	CNR1 rs6928499, rs1535255, rs2023239 polymorphisms	↓ risk metabolic syndrome in minor alleles carriers	Yu et al. (294)
SCZ	Blood	DNA genotyping	CB1r relative gene expression	↑ treatment response in G-allele carriers	Schennach et al. (233)
SCZ	Blood – PBMCs	Flow cytometry	CNR1 rs1049353 (1359 G/A) polymorphism	↑ CB1r	De Campos-Carli et al. (297)
SCZ	Blood - PBMCs	Quantitative polymerase chain reaction (qPCR)	CB1r expression	↑ CB1r	Chase et al. (298)
BD	Blood	DNA genotyping	CNR1 rs6454674 polymorphism	↑ severity in T/G heterozygotes	Alpak et al. (314)
Detroit Neighborhood Health Study (DNHS) participants	Blood	DNA genotyping	CNR1 rs1049353 polymorphism	↑ risk for PTSD-related symptoms in A-allele carriers with childhood abuse	Mota et al. (339)
PTSD	*In vivo* neuroimaging (several brain regions)	[^11^C]-OMAR PET	CB1r binding	↑ CB1r	Neumeister, A. et al. (342)
ADHD	Blood	DNA genotyping	SNP variants at the CNR1 gene	↑ frequency SNP haplotype (C-G)	Lu et al. (340)
ADHD (alcoholic patients)	Blood	DNA genotyping	CNR1 (AAT)n triplet polymorphism	↑ frequency longer form of alleles	Ponce et al. (358)
AN/BN	*In vivo* neuroimaging (several brain regions)	[^18^F]-MK-9470 PET	CB1r binding	↑ CB1r	Gérard et al. (371)
AN/BN	Blood	Quantitative polymerase chain reaction (qPCR)	CB1r relative gene expression	↓ CB1r in AN/BN women with self-injurious behavior	Schroeder et al. (372)
**CB2r**					
**Subjects/Diagnosis**	**Sample**	**Method**	**Measurement**	**Results**	**References**
General population	Buccal mucosa	DNA genotyping	CNR2 rs2501432 (R63Q) polymorphism	↑ risk for AD/DD in rs2501432 R-allele carriers with childhood trauma	Lazary et al. (176)
DD	Blood	DNA genotyping	CNR2 rs2501431 polymorphism	↑ depressive symptoms in G-allele carriers	Mitjans et al. (235)
DD	Blood	DNA genotyping	CNR2 rs2501432 (R63Q) polymorphism	↑ frequency R63Q polymorphism	Onaivi et al. (249, 241)
SCZ	Blood – PBMCs	Quantitative polymerase chain reaction (qPCR)	CB2r relative gene expression	↓ CB2r with clinical remission	De Marchi et al. (295)
SCZ	Blood – PBMCs	Quantitative polymerase chain reaction (qPCR)	CB2r relative gene expression	↑ CB2r	Chase et al. (298)
SCZ	Blood – PBMCs	Flow cytometry	CB2r expression	↑ CB2r	De Campos-Carli et al. (297)
SCZ	Blood – PBMCs	Western Blot	CB2r expression	↓ CB2r	Bioque et al. (310)
SCZ	Blood	DNA genotyping	CNR2 rs2501432 (R63Q), rs12744386 polymorphism	↑ frequency in rs2501432 R63 and rs12744386 C alleles	Ishiguro et al. (265)
SCZ	Blood	DNA genotyping	CNR2 rs2501432C/T polymorphism	↑ risk for SCZ in T-allele carriers	Tong et al. (311)
BD	Blood	DNA genotyping	CNR2 rs41311993 (524C/A) polymorphism	↑ frequency 524C/A polymorphism	Minocci et al. (317)

AD, anxiety disorder; DD, depressive disorder; SCZ, schizophrenia; FEP, first episode psychosis; BD, bipolar disorder; PTSD, post-traumatic stress disorder; ADHD, attention-deficit hyperactivity disorder; AN, anorexia nervosa; BN, bulimia nervosa; PMBT, post-mortem brain tissue; PET, positron emission tomography; PBMCs, peripheral blood mononuclear cells.

↓: decrease and ↑: increase.

**Table 2:** Main findings from human studies supporting the role of AEA and 2-AG as biomarkers in psychiatric disorders.

AEA & 2-AG					
Subjects/Diagnosis	Sample	Method	Measurement	Results	References
DD	Serum	Chemical ionization liquid chromatography-mass spectrometry (LC-APCI-MS)	AEA and 2-AG quantification	↑ AEA in minor depression ↓ 2-AG in major depression	Hill et al. (226)
DD	Serum	Chemical ionization liquid chromatography-mass spectrometry (LC-APCI-MS)	AEA and 2-AG quantification	↓ AEA ↓ 2-AG	Hill et al. (227)
DD	Plasma	Chromatography-coupled tandem mass spectrometry system	AEA and 2-AG quantification	↑ AEA	Romero-Sanchiz et al. (228)
DD	Serum	Electrospray ionization liquid chromatography-mass spectrometry (LC-ESI-MS-MS)	AEA and 2-AG quantification	↑ AEA = 2-AG after moderate exercise	Meyer et al. (230)
DD	CSF	Liquid chromatography-multiple reaction monitoring (LC/MRM)	AEA and 2-AG quantification	↑ AEA ↑ 2-AG after ECT	Kranaster et al. (231)
SCZ	CSF	High pressure liquid chromatography-gas chromatography/mass spectrometry (HPLC-GC/MS)	AEA quantification	↑ AEA	Leweke et al. (300)
SCZ	CSF	High pressure liquid chromatography-mass spectrometry (HPLC-MS)	AEA quantification	↑ AEA in antipsychotics-naïve patients = AEA in typical antipsychotics-treated patients	Giuffrida et al. (301)
SCZ	Blood	Chemical ionization liquid chromatography-mass spectrometry (LC-APCI-MS)	AEA quantification	↑ AEA ↓ AEA with clinical remission	De Marchi et al. (295)
SCZ	Plasma	Liquid chromatography-mass spectrometry (LC-MS)	AEA quantification	↑ AEA	Koethe, D. et al. (303)
SCZ	PMBT (several regions)	Liquid chromatography coupled with triple quadrupole mass spectrometry (LC/MS/MS)	AEA and 2-AG quantification	↓ AEA ↑ 2-AG	Muguruza et al. (306)
PTSD	Plasma	Chemical ionization liquid chromatography-mass spectrometry (LC-APCI-MS)	AEA and 2-AG quantification	= AEA ↓ 2-AG	Hill et al. (341)
AN	Plasma	Chemical ionization liquid chromatography-mass spectrometry (LC-APCI-MS)	AEA and 2-AG quantification	↑ AEA =2-AG	Monteleone et al. (370)

DD, depressive disorder; SCZ, schizophrenia; PTSD, post-traumatic stress disorder; AN, anorexia nervosa; CSF, cerebrospinal fluid; PMBT, post-mortem brain tissue; AEA, anandamide; 2-AG, 2-araquinoylglycerol.

↓: decrease and ↑: increase.

**Table 3:** Main findings from human studies supporting the role of FAAH as a biomarker in psychiatric disorders.

FAAH					
Subjects/Diagnosis	Sample	Method	Measurement	Results	References
Healthy controls	Blood	DNA Genotyping	FAAH rs324420 (C385A) polymorphism	= anxiety-related self-reports in A-allele and C/C genotypes	Gärtner et al. (171)
PING study participants	Saliva	DNA Genotyping	FAAH rs324420 (C385A) polymorphism	↓ anxiety level in A-allele adolescent carriers	Gee et al. (172)
Project FRONTIER participants	Blood	DNA Genotyping	FAAH rs324420 (C385A) & CRFR1 minor alleles polymorphisms	↑ anxiety level in FAAH A-allele and CRFR1 non-minors alleles carriers	Harris et al. (173)
Duke Neurogenetics Study (DNS) participants	Saliva	DNA Genotyping	FAAH rs324420 (C385A) and CRHR1 rs110402 polymorphisms	↑ risk for AD in FAAH A-allele and CRHR1 A-allele carriers	Demers et al. (174)
General population	Buccal mucosa	DNA Genotyping	FAAH rs324420 (C385A) polymorphism	↑ risk for AD/DD in A-allele carriers with childhood trauma	Lazary et al. (175)
General population	Buccal mucosa	DNA Genotyping	FAAH rs324420 (C385A) polymorphism	↑ risk for AD/DD in A-allele carriers with childhood trauma	Lazary et al. (176)
DD/BD	Blood	DNA Genotyping	FAAH rs324420 (C385A) polymorphism	↑ frequency AC genotype carriers	Monteleone et al. (232)
SCZ	PMBT – prefrontal cortex	Enzymatic assay – scintillation counting	FAAH activity	↑ FAAH activity	Muguruza et al. (273)
SCZ	Blood	Quantitative polymerase chain reaction (qPCR)	FAAH relative gene expression	↓ FAAH with clinical remission	De Marchi et al. (295)
BD	Blood	DNA Genotyping	FAAH rs324420 (C385A) polymorphism	↑ frequency AC genotype carriers	Monteleone et al. (232)

AD, anxiety disorder; DD, depressive disorder; SCZ, schizophrenia; BD, bipolar disorder; PMBT, post-mortem brain tissue; FAAH, fatty acid amido hydrolase.

↓: decrease and ↑: increase.

**Figure 2:**
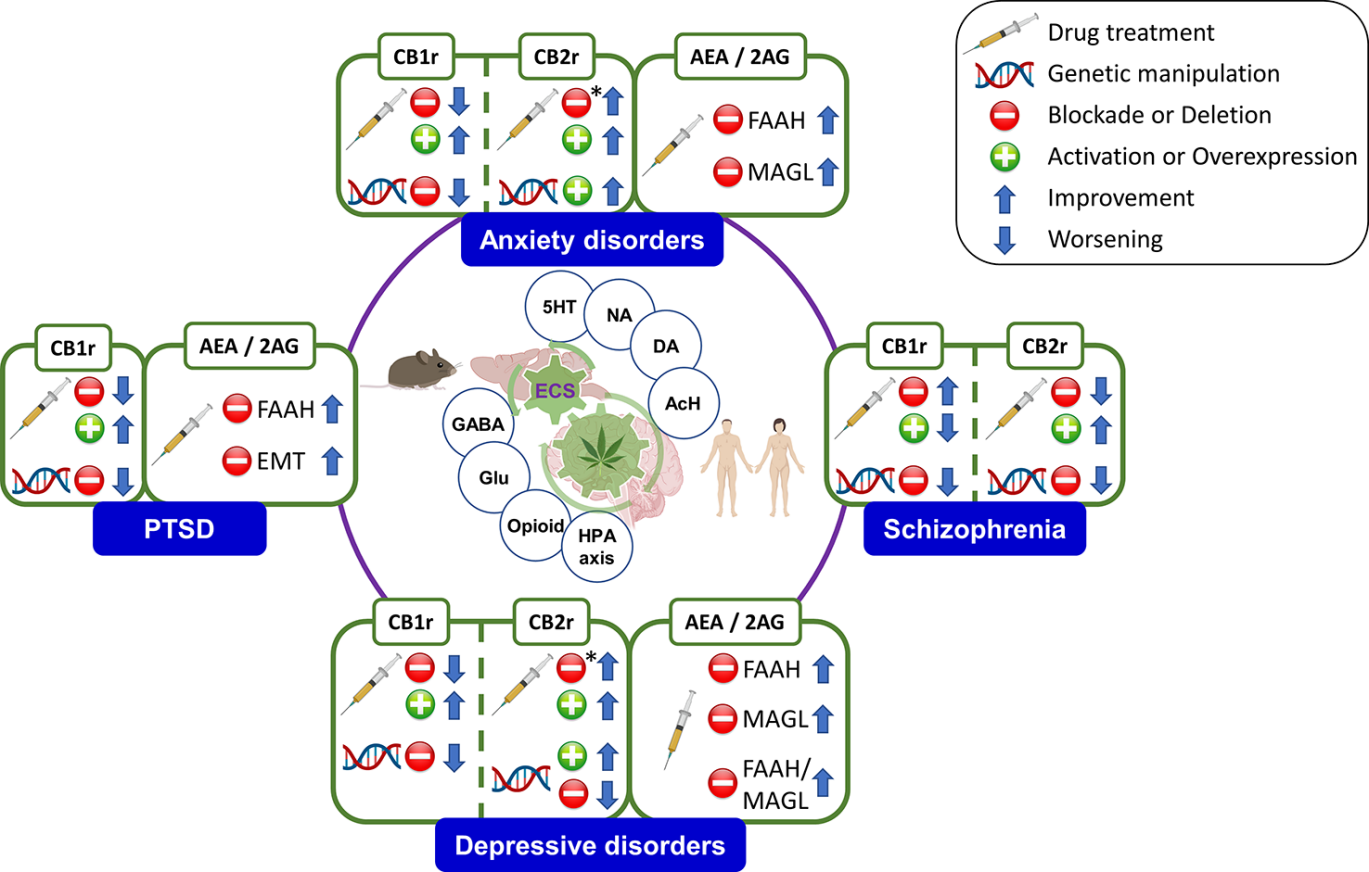
Main findings regarding the therapeutic potential of the functional manipulation of the endocannabinoid system (ECS) components by pharmacological and genetic approaches in anxiety, depression, schizophrenia, and post-traumatic stress disorder (PTSD). CB1r, cannabinoid receptor 1; CB2r, cannabinoid receptor 2; AEA, anandamide; 2-AG, 2-arachidonoylglycerol; FAAH, fatty acid amide hydrolase; MAGL, monoacylglycerol lipase; 5-HT, serotonin; NA, noradrenaline; DA, dopamine; AcH: acetylcholine; GABA, gamma-aminobutyric acid; Glu, glutamate; HPA axis, Hypothalamus–Pituitary–Adrenal axis; *, chronic treatment.

According to the information gathered in the present review from pharmacological and genetic approaches mainly performed in rodents, some general conclusions can be drawn regarding the usefulness of ECS components as therapeutic biomarkers (**Figure 2**). The blockade or genetic deletion of CB1r is closely associated with the worsening of emotional behavioral traits, as revealed principally in animal models of anxiety, depression or PTSD, whereas CB1r pharmacological activation induces an improvement effect. On the other hand, CB1r activation induces psychotic symptoms, while CB1r blockade presents antipsychotic effects. Regarding CB2r, its pharmacological activation or its overexpression by means of genetic manipulation or chronic treatment induced upregulation improving anxiety- and depressive-like behaviors, as well as schizophrenia-like traits. In contrast, all these behaviors worsen by CB2r blockade or gene deletion. Interestingly, the strengthening of the endocannabinoid tone, by the inhibition of enzymatic degradation or the blockade of reuptake mechanisms, is closely related with an improvement, particularly in emotional regulation as explored in animal models of anxiety, depression, and PTSD.

Therefore, the available evidence points out that the functional manipulation of the ECS components presents a great therapeutic potential. However, the close interaction of the ECS with other neurotransmitter or neurohormonal systems, as well as the specific and differential neuroanatomical distribution of the ECS components, provides a complex scenario not only from a therapeutic point of view but also considering the occurrence of side effects. In this sense, some aspects should be critically addressed, especially from a pharmacological perspective. The dosing, duration, and mechanism of action involved in the manipulation of the ECS are crucial to reach an improvement and limit adverse reactions. Apart from the widely explored role of CB1r, it should be noted that in recent years an increasing emphasis is being placed on the design of strategies to regulate endogenous cannabinoid tone, through inhibitors of the degradation or reuptake of eCBs. Since this approach provided negative results, particularly regarding the inhibition of FAAH (373), current trends focused on the combination of different mechanisms of action to enhance the endocannabinoid tone (374). Moreover, the pharmacological modulation of CB2r has also attracted much attention given its safety profile and the wide range of properties attributed to it, including mood and cognitive regulation. Thus, future priorities for both human and animal research would be the potentiation of both endocannabinoid tone and CB2r-mediated actions.

The trials carried out on patients show alterations of the ECS components at different levels, which in certain cases are related to risk or predictive factors regarding the evolution of the disease, or the degree of response to drug treatment. It is relevant to highlight the underlying sex-dependent effects in terms of sexual dimorphism of the ECS (375) and sex differences in prevalence rates and presentation of the psychiatric disorders (376). These could be involved not only in the changes of the ECS components to provide sex-related diagnostic or prognostic biomarkers, but also in the pharmacological actions derived from the treatment with cannabinoid compounds (377). Finally, it is important to note that more *in vivo* clinical studies are recently being carried out employing blood samples (PBMCs, plasma) or neuroimaging techniques (PET, fMRI) to identify ECS-related alterations, providing very relevant data. In this sense, a higher effort is required to design and perform more clinical studies, especially increasing the sample sizes to achieve greater significance, representativeness and reproducibility, finally making possible to identify some of the ECS components as useful biomarkers applicable to clinical practice in psychiatry.

## Author Contributions

FN and JM designed the sections and contents of the review manuscript. FN oversaw the organization to distribute the writing tasks among the authors and participated in manuscript writing. MG-G, RJ-B, GR, AG, and AA-O perform the literature searches and participated in the manuscript writing. All the authors critically reviewed and approved the final version of the manuscript.

## Conflict of Interest

The authors declare that the research was conducted in the absence of any commercial or financial relationships that could be construed as a potential conflict of interest.

## Abbreviations

ECS, endocannabinoid system; eCBs, endocannabinois; CB1r, cannabinoid receptor 1; CB2r, cannabinoid receptor 2; CSF, cerebrospinal fluid; PET, positron emission tomography; fMRI, functional magnetic resonance; BD, bipolar disorder; PTSD, posttraumatic stress disorder; ADHD, attention deficit hyperactivity disorder; YLDs, years lived with disability; DALYs, disability adjusted life-years; CNS, central nervous system; PFC, prefrontal cortex; NAc, nucleus accumbens; Hipp, hippocampus; Amy, amygdala; PVN, paraventricular nucleus; Hyp, hypothalamus; VTA, ventral tegmental area; AEA, anandamide; 2-AG, 2-arachidonoylglycerol; NAPE-PLD, N-acylphosphatidylethanolamine specific phospholipase D; FAAH, fatty acid amide hydrolase; GPCRs, Gq protein-coupled receptors; DAGL, diacylglycerol; MAGL, monoacylglycerol lipase; ABHD, alpha/beta-hydrolase domain; DSM-5, diagnostic and statistical manual of mental disorders, 5^th^ version; TRPV1, transient receptor potential cation channel subfamily V member 1; dlPAG, dorsolateral periaqueductal gray matter; NO, nitric oxide; WT, wild-type; DMH, dorsomedial hypothalamus; BLA, basolateral amygdala; EPM, elevated plus maze; LHb, lateral habenula; LC, locus coeruleus; DRN, dorsal raphe nucleus; NA, noradrenaline; 5-HT, serotonin; HPA axis, hypothalamus–pituitary–adrenal axis; CRH, corticotropin-releasing hormone; CRHR1, corticotropin-releasing hormone type 1 receptor; 5HTT, serotonin transporter; HDAC, histone deacetylase; CUS, chronic unpredictable stress; CB2xP, transgenic mice overexpression CB2r in the brain; BCP, beta-caryophyllene; THC, tetrahydrocannabinol; MDD, major depressive disorder; BDNF, brain-derived neurotrophic factor; TST, tail suspension test; FST, forced swimming test; CMS, chronic mild stress; FSL, Flinders sensitive line rats; WKY, Wistar Kyoto rats; cKO, conditional knockout; SSRIs, serotonin selective reuptake inhibitors; 5HTT, serotonin transporter; rTMS, repeated transcranial magnetic stimulation; ECT, electroconvulsive therapy; ACC, anterior cingulate cortex; PPI, prepulse inhibition; NMDAr, N-methyl-D-aspartate receptors; MAM, methylazoxymethanol; SHR, spontaneously hypertensive rat; CNR1P1, cannabinoid receptor interacting protein; Nrg1, neuregulin 1; DLPFC, dorsolateral prefrontal cortex; STG, superior temporal gyrus; qRT-PCR, quantitative real time polymerase chain reaction; FEP, first episode psychosis; PBMCs, peripheral blood mononuclear cells; PANSS, positive and negative syndrome scale; SUD, substance use disorder; CBD, cannabidiol; ACTH, adrenocorticotropin hormone; CS, corticosterone; SPS, single prolonged stress; DLS, dorsolateral striatum; PEA, palmitoylethanolamide; OEA, oleoylethanolamide; SEA, steraoylethanolamide; AN, anorexia nervosa; BN, bulimia nervosa; NPY, neuropeptide Y; POMC, proopiomelanocortin; CART, cocaine-amphetamine-regulated transcript; ARC, arcuate nucleus; FID, food intake disorders; BMI, body mass index.
